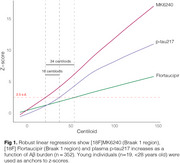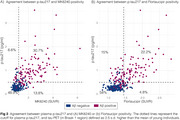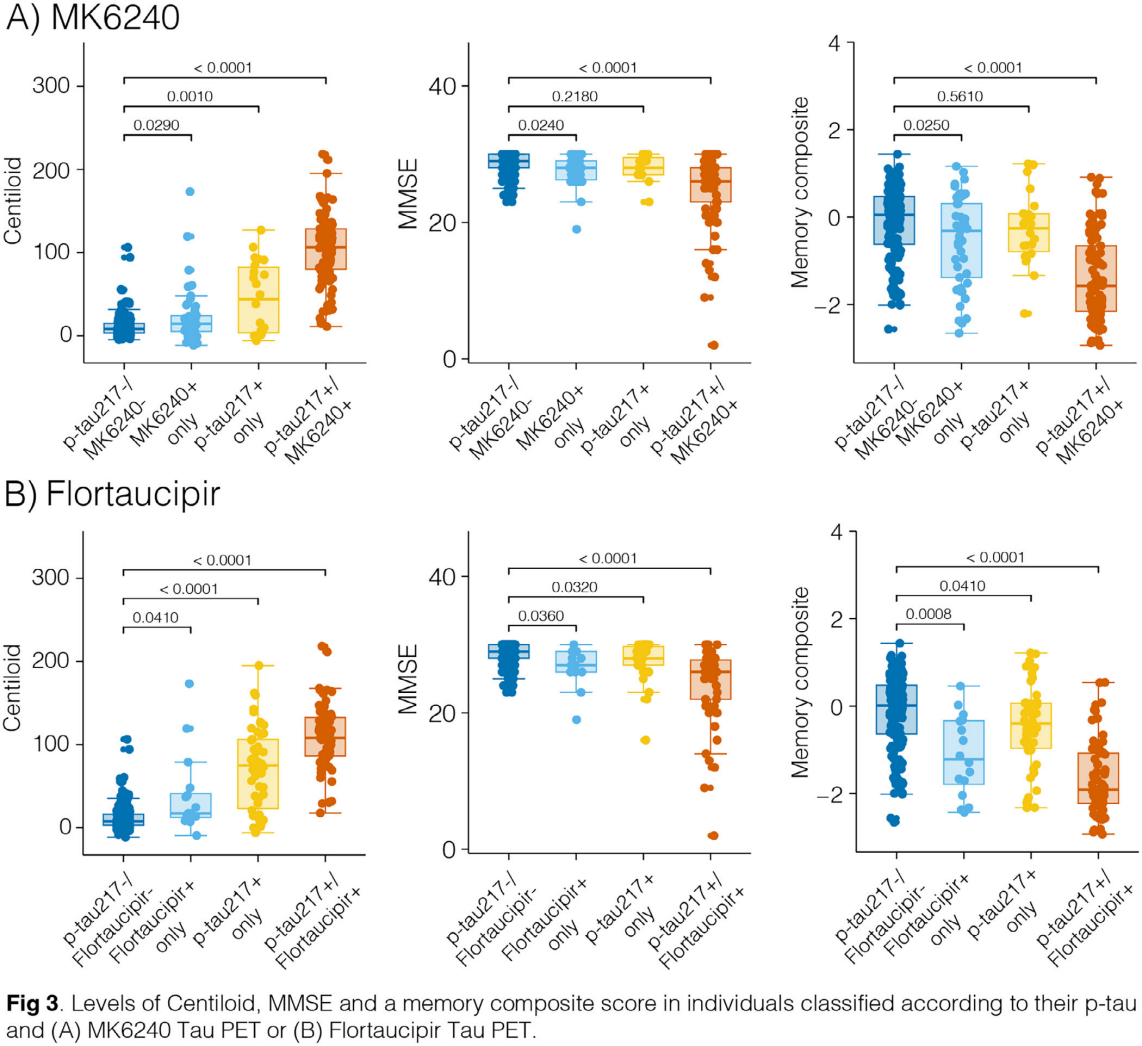# Head‐to‐head trajectories of MK6240, Flortaucipir, and plasma *p*‐tau217 as a function of Aβ

**DOI:** 10.1002/alz70862_110045

**Published:** 2025-12-23

**Authors:** Bruna Bellaver, Guilherme Povala, Pamela C.L. Ferreira, Guilherme Bauer‐Negrini, Firoza Z Lussier, Livia Amaral, Andreia Rocha, Carolina Soares, Joseph C. Masdeu, Dana L Tudorascu, Thomas K Karikari, David N. Soleimani‐Meigooni, Juan Fortea, Val J Lowe, Hwamee Oh, Belen Pascual, Brian A. Gordon, Pedro Rosa‐Neto, Suzanne L. Baker, Tharick A Pascoal

**Affiliations:** ^1^ University of Pittsburgh, Pittsburgh, PA USA; ^2^ Houston Methodist Research Institute, Houston, TX USA; ^3^ Memory and Aging Center, Weill Institute for Neurosciences, University of California San Francisco, San Francisco, CA USA; ^4^ Sant Pau Memory Unit, Department of Neurology, Institut d’Investigacions Biomèdiques Sant Pau ‐ Hospital de Sant Pau, Barcelona, Barcelona Spain; ^5^ Mayo Clinic, Rochester, MN USA; ^6^ Brown University, Providence, RI USA; ^7^ Washington University in St. Louis, School of Medicine, St. Louis, MO USA; ^8^ McGill University, Montreal, QC Canada; ^9^ Lawrence Berkeley National Laboratory, Berkeley, CA USA

## Abstract

**Background:**

Tau PET tracers present distinct binding characteristics that might influence their trajectories and relationship with other biomarkers along the AD continuum. In a head‐to‐head study, we investigated the relationship between the emergence of PET tracers MK6240 and Flortaucipir, and plasma *p*‐tau217 abnormalities as a function of Aβ PET deposition. We further assessed the concordance between tau PET and plasma *p*‐tau217 positivity and its relationship with cognitive scores.

**Method:**

We evaluated 352 individuals from the HEAD study (205 cognitively unimpaired and 147 cognitively impaired) with Aβ PET, MK6240 and Flortaucipir tau PET, and plasma *p*‐tau217 (ALZpath) and GFAP (Quanterix) measures. Tau PET Braak regions, plasma *p*‐tau217 trajectories were modeled as functions of Aβ burden (Centiloid scale) using the Lowess method. Biomarkers were z‐scored anchored on young individuals (*n* = 19, < 28 years old) as anchors. Tau PET (Braak 1 region) and plasma *p*‐tau217 were considered positive/abnormal when surpassing 2.5 z‐score.

**Result:**

Among the tested markers, MK6240 was the earliest to show abnormality as a function of Centiloid, occurring at 22 Centiloids, followed by plasma *p*‐tau217 at 38 Centiloid and Flortaucipir at Centiloid 56 (Figure 1). Tau PET and plasma *p*‐tau217 positivity showed an overall high concordance (∼80% for both tracers; Figure 2). In MK6240 discordant cases, most individuals were MK6240+/*p*‐tau217‐ (13.8%), while 6.6% were MK6240‐/*p*‐tau217+ (Figure 2A). For Flortaucipir, 15% of discordant cases were Flortaucipir‐/*p*‐tau217+, while 4.8% were Flortaucipir+/*p*‐tau217‐ (Figure 2B). The discordant groups present higher Centiloid than the ptau217‐/Tau‐ group and overall decreased scores in cognitive tests, except for *p*‐tau217+/MK6240‐ group (Figure 3).

**Conclusion:**

MK6240 becomes abnormal at lower levels of Aβ burden compared to plasma *p*‐tau217 and Flortaucipir. The relatively high prevalence of discordant tau PET positive or plasma *p*‐tau217 positive and suggests that some individuals may show tau PET positivity first, while others may exhibit plasma *p*‐tau217 positivity first. The distinct cognitive profiles of this groups suggest potential clinical relevance, warranting further investigation.